# EEG features and synek scale indicate severity of neurotoxicity in adult patients treated with CD19 CAR T-cell therapy

**DOI:** 10.1038/s41598-024-80566-0

**Published:** 2024-11-23

**Authors:** David Mao, Anne S. Reiner, Xi Chen, Jae Park, Martina Pennisi, Miguel-Angel Perales, Edward K. Avila, Bianca D. Santomasso

**Affiliations:** 1 Department of Neurology, Memorial Sloan Kettering Cancer Center, New York, NY USA; 2 Department of Epidemiology and Biostatistics, Memorial Sloan Kettering Cancer Center, New York, NY USA; 3 Adult Bone Marrow Transplantation Service, Department of Medicine, Memorial Sloan Kettering Cancer Center, New York, NY USA; 4 Division of Hematology and Stem Cell Transplantation, Fondazione IRCCS Istituto Nazionale dei Tumori, Milan, Italy; 5 Department of Medicine, Weill Cornell Medical College, New York, NY USA; 6 Memorial Sloan Kettering Cancer Center, 1275 York Avenue, New York, NY 10065 USA

**Keywords:** ICANS, CAR T-cell therapy, EEG, Synek, Immune-related adverse events, Cancer, Biomarkers, Medical research, Neurology, Oncology, Pathogenesis

## Abstract

Patients who develop chimeric antigen receptor (CAR) T-cell-related immune effector cell-associated neurotoxicity syndrome (ICANS) frequently undergo evaluation with electroencephalography (EEG). We hypothesize that EEG features and Synek scale score, a measure of degree of EEG abnormality, are associated with ICANS severity. Here, we performed a retrospective review of 125 adult patients at Memorial Sloan Kettering Cancer Center (MSKCC) who received CAR-T cell therapy from 2010 to 2019, including 53 patients with B-acute lymphoblastic leukemia treated with 1928z CAR T cells (NCT01044069) and 72 patients with large B-cell lymphoma (LBCL) treated with the commercial CAR T products axicabtagene ciloleucel or tisagenlecleucel. We collected video EEG monitoring (27 with B-ALL and 20 with LBCL) and recorded daily EEG features, Synek scores, and ICANS grade for 47 eligible patients. Synek scale and ICANS grade were positively correlated (correlation coefficient 0.47, 95% CI: 0.31–0.60). This was further corroborated in the univariable model associating high Synek scale (3 or 4) with high ICANS grade (OR = 15.2; 95%CI:7.8–29.7, *p* < 0.0001). EEG features such as discontinuity, absence of posterior dominant rhythm, and presence of generalized sharp waves were statistically significantly associated with higher ICANS grade in univariable models. In the multivariable model, discontinuity (OR = 4.2 (95%CI:1.3–13.8, *p* = 0.02) and absence of posterior dominant rhythm (OR = 10.5 (95%CI:4.6–23.9, *p* < 0.0001) were statistically associated with higher ICANS grade. Overall, EEG discontinuity and absence of posterior dominant rhythm were independently associated with higher severity of neurotoxicity. Further, our data suggest that Synek Scale, may be a severity marker for neurotoxicity.

**Supplementary Information:**

The online version contains supplementary material available at 10.1038/s41598-024-80566-0.

## Introduction

Chimeric antigen receptor (CAR) T cell therapy has revolutionized the prospects and therapeutic options for some hematological malignancies, achieving durable clinical responses in high-risk patients such as those with relapsed and refractory malignancies including B-cell acute lymphoblastic leukemia and lymphoma^1–4^. There are now six commercially available FDA-approved CAR T-cell therapies for hematologic malignancies. Despite promising efficacy signals, acute toxicities of CAR T cell treatment, including cytokine release syndrome (CRS) and immune effector cell associated neurotoxicity syndrome (ICANS), remain a challenge and raise serious concerns about their potential for broad use and applicability^5^. ICANS occurs in over 60% of adults treated for aggressive leukemias or lymphomas, with up to 30% of patients experiencing grade ≥ 3^6^. It typically manifests as generalized encephalopathy with confusion and behavioral changes and/or language impairment^7^. Other symptoms include tremor, myoclonus, seizures, status epilepticus, somnolence, and coma and, more rarely, cerebral edema and death^8–11^.

The neurological symptoms of ICANS were identified in the earliest CAR T clinical trials almost two decades ago, but consensus grading and terminology was only established in 2019^12^. Despite a better understanding of the clinical features of ICANS, the pathophysiologic basis of CAR T neurotoxicity remains under investigation. Inflammatory markers and cytokines have been associated with increased ICANS severity, but are non-specific and also elevated during CRS^9^. Corticosteroids remain the main treatment for ICANS and works to limit inflammatory process in the central nervous system. Supportive care such as seizure control and management of cerebral edema is also critical^10^. Anakinra (IL1Ra antagonist) is also used for the mitigation of severe ICANS symptoms in patients treated with CAR T cells^13^. Given the treatment options, accurate and timely diagnosis and grading of ICANS is vital to management of these serious immune-mediated adverse events, as rapid clinical progression necessitating critical care may occur. Thus, there is an urgent and unmet need for the development of severity markers of ICANS to predict and monitor its occurrence and duration, and to evaluate responses to experimental ICANS prophylactic and therapeutic interventions.

For patients who develop ICANS, an electroencephalography (EEG) is an essential tool to diagnose non-convulsive seizures. EEG features such as diffuse slowing, discontinuity, and absence of posterior dominant rhythm (PDR) which can be used to measure degree of encephalopathy and prognosticate outcomes in Intensive Care Unit (ICU) patients^14^. These EEG features are utilized in Synek scale, which quantifies EEG abnormalities and provides a severity grade. Prior studies have suggested that EEG findings may be severity markers for ICANS. In one study analyzing EEG patterns in children and young adults treated with CAR T-cell therapy, the authors found that Synek scale was better able to differentiate ICANS severity compared to clinical scores^15^. Similar study of adult patients treated with CAR T cell therapy also found correlation between Synek scale and peak neurotoxicity^16,17^.

In the present study, we aimed to describe daily EEG features, using the largest possible EEG dataset in two large cohort of patients with B-ALL and LBCL that had been treated with CAR T cells. We observe that certain EEG features are associated with higher ICANS grade. Overall, our data supports the hypothesis that the Synek scale provides a minimally invasive tool to assess daily ICANS severity in adult patients with hematological malignancies treated with CAR T cells.

## Methods

### Patients and ethics declaration

This retrospective analysis included adult patients who received CD19 CAR-T cell therapy, including patients with B-cell acute lymphoblastic leukemia (B-ALL) enrolled at our center in a phase 1 trial of 1928z CAR T cells from May 2010 through August 2016 (NCT01044069)^18^ and consecutive patients with non-Hodgkin large B-cell lymphoma (LBCL) treated out our center with commercially available axicabtagene ciloleucel or tisagenlecleucel, starting after US Food and Drug Administration approval from February 2018 to September 2019. Patients were included in our dataset if they were monitored on 24-hour continuous video electroencephalography (vEEG) after cell infusion. Additionally, the vEEG studies must have been completed during the same admission of CAR T-cell treatment and the indication of the study must have been for the medical management of ICANS.

All patients signed statements of informed consent under protocols approved by the MSKCC Institutional Review Board. The study was conducted in accordance with the Declaration of Helsinki, and informed written consent was obtained from each participant. Data on patient seizures is provided within the supplementary information files. Detailed data include personal information that cannot be shared openly to protect study participant privacy.

## Review of EEGs

EEG studies were reviewed by neurologists who are board certified in clinical neurophysiology. We reviewed EEG reports and recorded features of EEG including background abnormalities, focal findings, generalized sharp waves with triphasic morphology (“triphasic waves”), epileptiform activity, and seizures.

Features were reported using the 2016 ACNS Guidelines for EEG reporting as well as Critical Care terminology^19^. We also calculated Synek scale (Table 1) using the collected data^14^. Normal EEG background was defined as organized (consisting of an anterior-posterior frequency gradient), continuous, with a posterior dominant rhythm (PDR) of 8.5–12 Hz. A discontinuous background was described as a pattern of attenuation/suppression alternating with higher voltage activity, with 10–49% of the record consisting of attenuation or suppression^19^. Slower PDR frequencies were considered if they were in the theta (4–7 Hz) or delta (1–4 Hz) for a given state. Absence of the PDR was considered an abnormal finding^19^. Generalized slowing was defined as theta and delta frequencies during awake states and taken in context for drowsy and sleep states. Spikes, polyspikes, and sharp waves were defined as described by Kane et al. in 2017^20^. Sharp waves were interictal epileptiform discharges (IED), which were markers of increased cortical irritability and indicated underlying increased seizure potential, either focal or generalized. Triphasic waves were reported as described by Kane in 2017 and were a sign of diffuse cerebral dysfunction due to a metabolic etiology^20^. We defined “abnormal PDR” as PDR with frequency of less than or equal to 8.5 hz.

**Table 1:** Synek Scale grading^18^

Grade 0	Normal
Grade 1	There is an organized posterior dominant rhythm (PDR) with normal frequency and rare theta slowing.
Grade 2	The PDR is slow and not necessarily present. Background is mostly theta frequency.
Grade 3	The PDR is absent and background is mostly delta frequency.
Grade 4	The PDR is absent and background is slow with frequent discontinuity or burst suppression
Grade 5	Background is characterized by voltage suppression < 5 µV

## CRS and ICANS

Demographic and clinical data were retrospectively extracted from the electronic medical record. CRS and ICANS were graded according to the American Society for Transplantation and Cellular Therapy (ASTCT) grading system^12^ and were considered high grade if grade ≥ 3. CRS and ICANS data were collected daily in both cohorts; we collected both parameters prospectively for patients with LBCL and were confirmed by retrospective review. CRS and ICANS grading weas assigned retrospectively for the B-ALL cohort^8^. Since the B-ALL cohort was treated before the introduction of the ASCTC grading, a chart review was conducted to retrospectively grade CRS and ICANS, as previously described^8,21^. At our institution, neurological exams are conducted daily as part of the standard of care, and thus ICANS gradings were accordingly collected daily in both cohorts.

## Statistics

We used a repeated measures univariable generalized estimating equation (GEE) with logit link and exchangeable covariance matrix to find association of each EEG finding as well as Synek score with high neurotoxicity grade. No multiple testing adjustments were performed due to the small number of associations tested. Variables which were statistically significant univariably were entered into a multivariable GEE model. Synek score was deliberately not entered into the multivariable model as it is a composite of other EEG findings. In a post-hoc analysis, the multivariable model was further adjusted for cefepime and imipenem use. Synek score and ICANS grade were correlated using a repeated measures correlation coefficient with the R package rmcorr. The sensitivity and specificity of binary Synek score (grade 3, 4 vs. 0, 1, 2) against a gold standard of high ICANS (grade 3, 4 vs. 1, 2) was estimated with corresponding 95% CIs accounting for clustering within patient. Peak Synek score was associated with survival using Cox regression. Follow-up time was calculated from treatment start date until death due to treatment for those with events or until last known follow-up date for those who were censored. Tests were two-sided and statistical significance was set at < 0.05. Analyses were performed in SAS, v9.4 (The SAS Institute, Cary, NC) and R, v4.1.3 (The R Foundation for Statistical Computing).

## Results

### Patient demographic and clinical findings

We assembled two retrospective case series of 125 patients in total, encompassing 53 patients with B-cell acute lymphoblastic leukemia (B-ALL) treated with 19-28z CAR T cells [NCT01044069], ^16^ and 72 patients with Large B-Cell Lymphoma (LBCL) treated with axicabtagene ciloleucel or tisagenlecleucel at MSK. 50% (62 of 125) of the overall population developed ICANS of any grade, similarly to each individual cohort, with 62% (33 of 53) of B-ALL patients and 40% (29 of 72) of LBCL patients developing ICANS. Of the 62 patients who developed ICANS, we identified a total of 47 patients (27 with B-ALL and 20 with LBCL) who had vEEG studies that met the inclusion criteria (See Table 2). Patients were excluded if they lacked vEEG data (i.e., cases where only routine EEG was obtained) or if the EEG was not done during the period where ICANS symptomatology was observed.

**Table 2:** Patient Demographics and clinical summary for CAR T Cell patients with video EEG.

Patient characteristics	Overall population	B-ALL patients	LBCL patients
Patients, n	125	53	72
Patients with vEEG during toxicity	48	27	20
Median age in years (range)	57 (20–77)	37 (24–68)	69 (20–77)
Sex, n (%)			
Female	11 (23%)	6 (22%)	5 (24%)
Male	37 (77%)	21 (78%)	15 (76%)
Days of vEEG monitoring, n	15	110	47
Median number of days of vEEG monitoring per patient (Range)	2 (1–21)	2 (1–21)	2(1–7)
Number of patients with high grade neurotoxicity	30 (65%)	20 (78%)	10 (48%)
Number of patients required intubation	10 (20%)	7 (26%)	3 (14%)

**Fig 1:**
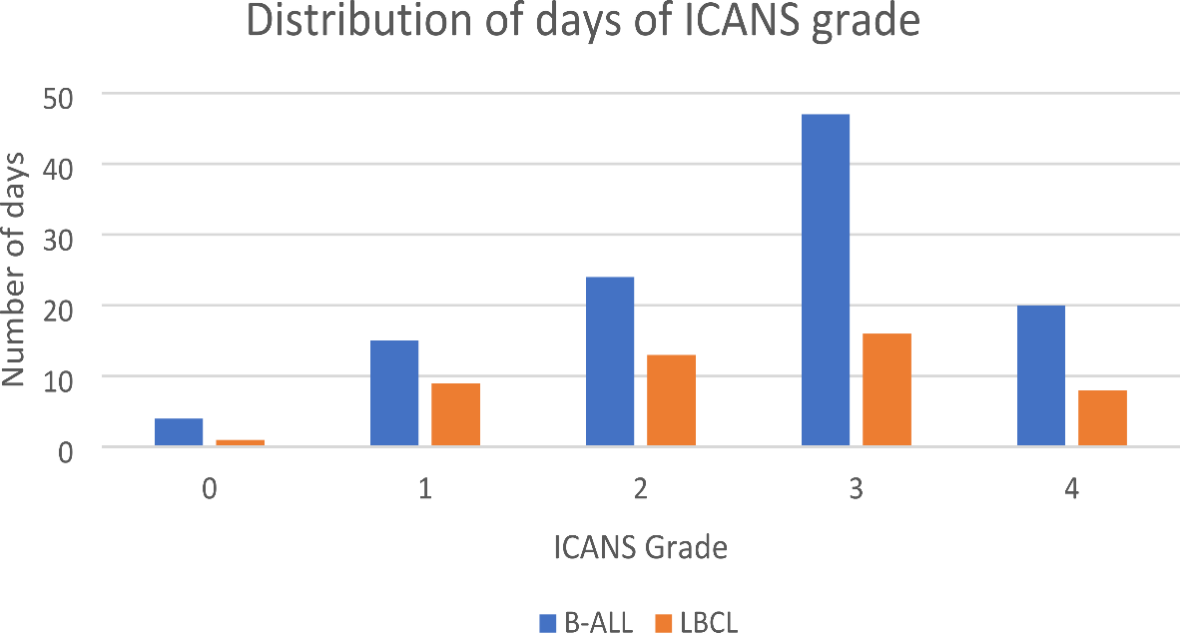
Displayed is the number of days on vEEG monitoring against each ICANS grade, split into B-ALL (blue bars) and LBCL cohorts (orange bars).

For the whole cohort, there was a total of 157 days of paired vEEG monitoring and ICANS assessments, 110 days in the B-ALL group and 47 days in the LBCL group. Patients had vEEG monitoring for a median of 2 days (range 1 to 21) days. This combined vEEG data set included 5 days of vEEG with grade 0 ICANS, 22 days of grade 1, 37 days of grade 2, 67 days of grade 3, and 26 days of grade 4, with similar distribution bins between B-ALL and LBCL (Fig. 1). During their vEEG monitoring period, 4 patients had peak ICANS grade of 4, 26 had grade 3 ICANS, 8 patients had grade 2 ICANS, and 9 had grade 1 ICANS. Combined, 64% of the patients included in the study developed severe ICANS. All patients with ICANS experienced CRS.

None of the patients had prior history of epilepsy. MRI brain with and without contrast performed after onset of encephalopathy did not reveal any acute findings that could explain findings on vEEG. In the B-ALL group, one patient had incidental left frontal petechial hemorrhage. Another had a 0.5 cm chronic subdural hematoma. As previously reported, 4 patients developed a pattern of T2/FLAIR hyperintensities involving the bilateral thalami and brainstem that reversed with resolution of ICANS^18^. In the LBCL cohort, one patient had incidental finding of enlarged ventricles, and another had enhancing lesions in the left thalamus likely indicative of central-nervous system dissemination of the tumor.

All patients enrolled after 2013 received levetiracetam 500 mg twice a day for seizure prophylaxis, prior to CAR T cell infusion. Additional antiseizure medications were added and titrated based on EEG findings, including Levetiracetam, Phenytoin, Lacosamide, Lorazepam, Phenobarbital, and other anesthetic agents depending on clinical context. Patients received Dexamethasone or Tocilizumab based on hospital protocol for treatment of CRS and ICANS. Levetiracetam prophylaxis, when given, was started on or before the day of CAR T-cell infusion. 7 patients that were enrolled from 2012 to 2013 did not receive levetiracetam prophylaxis. Of these, 3 ultimately had generalized tonic-clonic seizures. The full list of patients with clinical events suspicious for seizures are described in Supplemental Tables 2 and 3.

## Distinct EEG features

**Fig 2:**
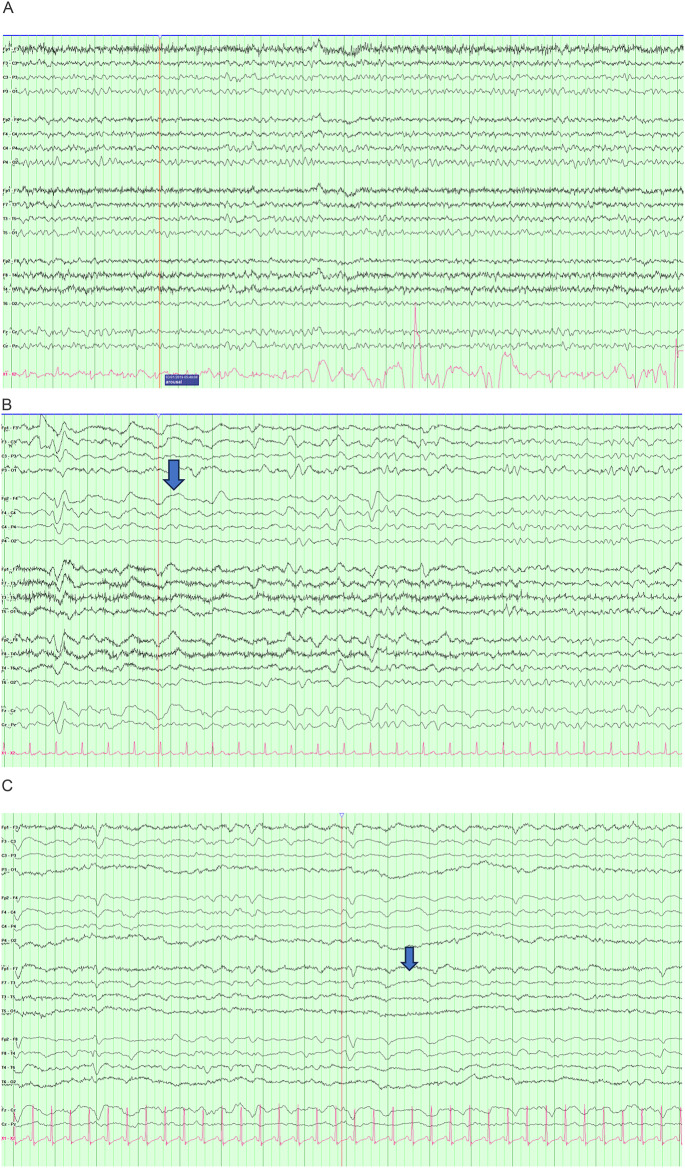
Sample EEG: Time base: 15mm/second, Sensitivity: 7 uV/mm. (**A**) EEG with Synek score of 1. Patient is 73-year-old man with DLBCL and peak ICANS of 2. Note organized background with normal PDR and occasional slowing. (**B**). Synek score of 3. Patient is 70-year-old woman with DLBCL and peak ICANS grade of 3. Note near continuous delta activity and lack of organized rhythm. (**C**). Synek score of 4. This is 34-year-old woman with B-cell ALL with peak ICANS grade of 4. Note the discontinuous background and delta slowing.

We identified patients with specific EEG features, including the absence of posterior dominant rhythm, discontinuity, diffuse slowing, rhythmic slowing, focal slowing, triphasic waves, generalized sharp waves, focal sharp waves, and seizures (Table 3). For posterior dominant rhythm, we characterized it as present or absent. If a posterior dominant rhythm was present, we further characterized it as abnormal if the frequency is less than or equal to 8.5 hz. (See Fig. 2 for examples). For focal slowing and focal sharp waves, one patient had bilateral independent focal slowing and one patient had multifocal sharp waves.

**Table 3:** Number of patients with each EEG feature over the course of monitoring.

	B-ALL	LBCL	Overall	Comment
PDR^$^				
Absent	**12 (44%)**	**7 (35%)**	**19 (40%)**	
Present	**15 (56%)**	**13 (65%)**	**28 (60%)**	
And normal	**2**	**3**	**5**	
And abnormal	**13**	**10**	**23**	
Discontinuous background	**8 (30%)**	**4 (19%)**	**12 (25%)**	
Focal Slowing	**5 (19%)**	**4 (19%)**	**9 (19%)**	*One patient had bilateral focal slowing
Left Hemisphere	3*	**4**	**7**	
Right Hemisphere	3*	**0**	**3**	
Diffuse slowing	**27 (100%)**	**20 (100%)**	**47 (100%)**	
Rhythmic delta activity	**9 (33%)**	**4 (19%)**	**13**	
Focal sharp waves	**3 (11%)**	**2 (10%)**	**5 (10%)**	*One patient had multifocal sharp waves
Left Hemisphere	**1**	2*	**2**	
Right Hemisphere	**2**	1*	**3**	
Generalized sharp waves	**3 (11%)**	**2 (10%)**	**5 (10%)**	
Triphasic waves	**6 (22%)**	**7 (10%)**	**13 (27%)**	
With eventual seizures	**4**	**0**	**4**	
Seizures*	**7 (26%)**	**1 (5%)**	**8 (17%)**	*Only includes clinical events captured on vEEG
Generalized	**1**	**1**	**2**	
Focal	**4**	**0**	**4**	
Scalp negative events	**2**	**0**	**2**	

^$^We defined “abnormal PDR” as PDR with frequency of less than or equal to 8.5 hz.

In the B-ALL group, 3 patients displayed focal interictal sharp waves, including over the left central region, right frontal region, and right posterior quadrant region with clinical correlate. In the LBCL group, there was one patient with left centroparietal sharp waves and another with multifocal sharp waves. 6 patients with B-ALL had generalized sharp waves with triphasic morphology (triphasic wave). The LBCL cohort also had high number of patients with triphasic waves at 7.

We entered these variables into the multivariable model and obtained results shown in Table 4. In the multivariable model, absence of posterior dominant rhythm and discontinuous background were independently, statistically significantly associated with high neurotoxicity grade. This was true even after further adjustment for cefepime and imipenem use in a post-hoc analysis. We found that absence of PDR, discontinuity, and generalized sharp waves had statistically significant associations with high grade ICANS.

**Table 4:** Multivariable associations between EEG findings and high neurotoxicity grade.

vEEG features	Odds ratio (95% Confidence Interval)	*P*-value
Absence of PDR	10.5 (4.6–23.9)	< 0.0001
Discontinuity	4.2 (1.3–13.8)	0.02
Generalized sharp waves	4.5 (0.5–38.5)	0.17

## Seizures correlate with vEEG events

Seizures are defining features of high grade ICANS^12^. Different seizure types were observed through daily vEEG recording (Table 3). In the B-ALL group, 7 patients had at least one suspicious event captured on vEEG. In this group, 5 patients had electrographic seizures, while 2 had clinical events that did not have EEG correlate. There were four patients with focal onset seizures and one with generalized onset. In the LBCL group, one patient experienced a seizure event that was captured on vEEG and which showed generalized periodic sharp waves.

Additional patients had clinical events that were not captured on vEEG (Supplemental Tables 2 and 3). We summarize the clinical and vEEG findings of patients in both B-ALL and LBCL who had clinical events that were suspected as seizures by the clinical team. Because vEEG was connected after the reported events and escalation in treatment, not all patients would later have additional events that could be captured on vEEG. For this reason, we included brief description of the EEG findings and the specific event that prompted suspicion of seizures.

The B-ALL group had 18 had clinical events that were concerning for seizures as defined above (Supplemental Table 2), while the LBCL group had 5 (Supplemental Table 3). Clinically, their symptoms included myoclonic jerks, episodic aphasia, and confusion. 6 of these patients progressed to witnessed generalized tonic-clonic (GTC) seizures. In B ALL group there were 3 patients with non-convulsive status epilepticus. In the LBCL there was 1 patient (Supplemental Tables 2 and 3). Among the patients with LBCL, there was lower percentage of patients with high grade ICANS (Table 2), which could explain the lower portion of patients with suspected seizure events. The five patients with suspected seizures in LBCL group had similar mixture of symptoms.

Certain antibiotics are known to lower seizure threshold. During the EEG monitoring days, in the LBCL group, 3 patients received cefepime and one had seizures during EEG monitoring. In the ALL group, 8 patients received cefepime and 5 had seizures. Additional 6 patients in the ALL group received imipenem and 1 had seizures. 1 patient in the ALL group received both cefepime and imipenem, and they also had seizures on EEG.

### Synek scale modeling correlates with ICANS grade

The Synek scoring scale is defined in Table 1^14^. We calculated Synek scale using our retrospectively collected, daily EEG data. The results of our univariable and multivariable models appear in Tables 5 and 4, respectively. An odds ratio (OR) > 1.0 is associated with an increased risk of high grade ICANS (grade 3 or 4). An OR < 1.0 is associated with a decreased risk of high grade ICANS (grade 3 or 4).

**Table 5:** Unadjusted associations between EEG findings and high ICANS Grade.

vEEG features	Odds ratio (95% Confidence Interval)	*P*-value
Absence of PDR	14.9 (7.2–30.9)	< 0.0001
Discontinuity	13.0 (4.6–37.2)	< 0.0001
Diffuse slowing	Did not converge	--
Focal slowing	1.7 (0.9–3.3)	0.14
Focal sharp waves	1.8 (0.6–5.4)	0.29
Triphasic wave	2.6 (0.8–8.9)	0.12
Generalized sharp waves	3.0 (1.5–5.9)	0.0018
Rhythmic delta activity	0.9 (0.4–2.1)	0.77

**Fig 3:**
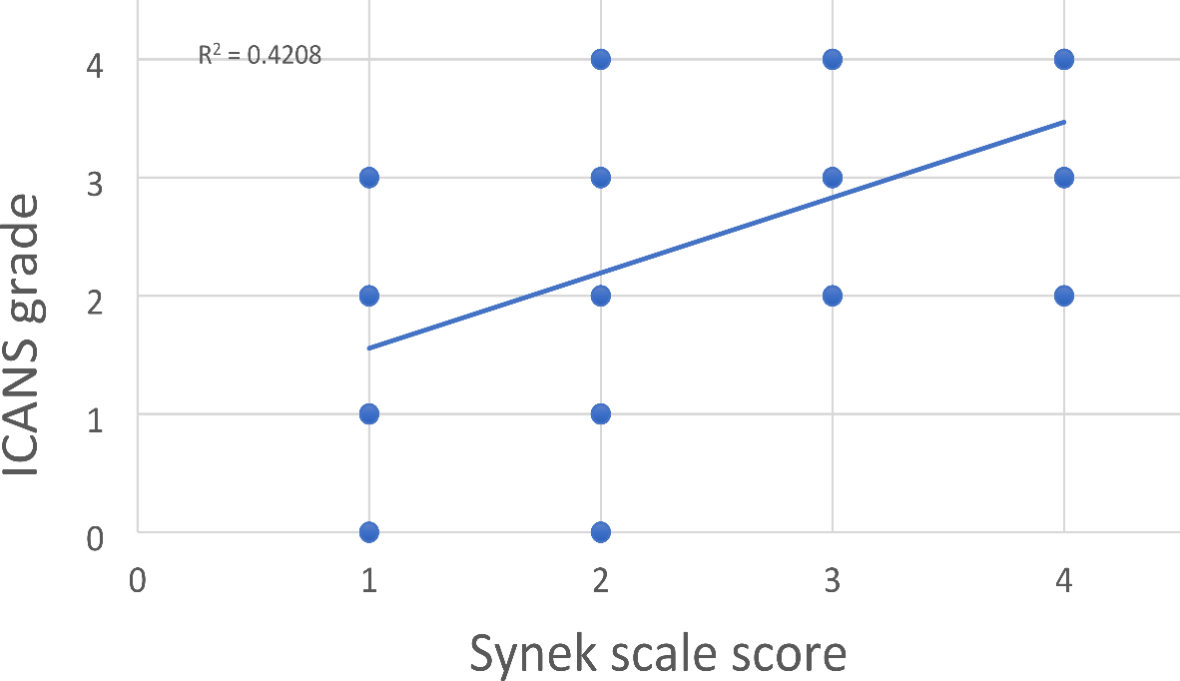
Continuous Synek scale was positively correlated with continuous ICANS grade.

Synek scale was highly associated with high ICANS grade. When dichotomized, high Synek score (Grade 3 or greater) was associated with a 15-fold risk of having high ICANS grade (OR = 15.2; 95%CI:7.8–29.7, *p* < 0.0001). A similar trend was seen with each increasing point on the Synek scale. The continuous Synek scale was positively correlated with continuous ICANS grade (repeated measure correlation coefficient 0.47, 95% CI: 0.31–0.60)). Scatter plot of this relation can be seen in Fig. 3. The sensitivity of Synek score (grade 3, 4 vs. 0, 1, 2) to designate a patient as high ICANS was 68.1% (95% CI: 50.7-82.5%) and the specificity to designate a patient as low ICANS was 87.5% (95%CI: 76.8-94.4%).

We also performed survival analysis to identify if Synek scale can predict survival. We did not identify an association between Synek score and overall survival using continuous Synek score (hazard ratio: 1.0, 95% CI: 0.7–1.4) or dichotomized to low (0, 1, or 2) and high grade (3 or 4) Synek score (hazard ratio: 1.1, 95% CI: 0.6–2.3).

## Discussion

We used a statistical model to study the relationship between daily EEG features and ICANS grade. This type of analysis has only been done recently and our data adds to the growing literature around neurotoxicity in CAR T cell therapy and the utility of daily EEG monitoring^17^. While we had separate patient populations with B-ALL and LBCL, the primary analyses were performed in the overall cohort (combined patients with B-ALL and LBCL) to provide a comprehensive overview of the association between daily EEG features and Synek score with ICANS grade using the largest possible sample size. Statistical analysis of EEG features showed absence of posterior dominant rhythm, seizures, and discontinuity to be associated with higher grade neurotoxicity.

Our study adds to the growing evidence that EEG background can be scored for use in evaluating ICANS. However, there was no relation found to long term survival. Similar findings were reported in one paper that studied a cohort of children and young adult patients with pediatric B cell malignancies treated with CAR T-cell therapy. It found that worsening severity of EEG background correlates with worse ICANS^15^. Another paper found correlation between EEG captured on day of peak ICANS and Synek but did not evaluate this relationship over the course daily EEG’s^16^. In addition, recent study by Jones et al., the authors found a correlation between slowing of EEG features (by measuring 15-second EEG clips over a 24-hour period) and ICANs, using machine learning models^17^. In comparison, our study was conducted with longitudinal EEG scoring through daily vEEG monitoring over 1–21 days in two independent cohorts of patients treated with CAR T cell products. Our study provides a proof-of-concept feasibility in clinical settings devoid of computations or machine learning experts. Although Synek scale is not a diagnostic marker, we incorporate this into our proposed prognostic pipeline to assess its correlation with ICANS severity through the development of Synek scores. Further studies are needed to evaluate the predictive power and accuracy of Synek scores in ICANS severity measure and prognosis, and as a tool for monitoring daily changes in ICANS. Our data supports the use of Synek scores in adults for ICANS monitoring, complementing standard clinical evaluation.

Synek scale incorporates generalized slowing, which is defined as theta and delta frequencies during awake states. Normal 8.5–12 Hz posterior dominant rhythm is expected in healthy individuals. Conversely, slower posterior dominant rhythm or its absence can be a sign of diffuse and non-specific cerebral dysfunction. Generalized slowing is a non-specific sign of diffuse cerebral dysfunction and has been associated with toxic-metabolic, post-ictal, or increased intracranial pressure^22^. It can be a sign of acute or subacute evolving underlying disturbance. Further, slowing can be rhythmic, forming rhythmic delta activity. Recently, an EEG-based grading scale was developed that correlated with ICANS severity with lower frequencies and even changes of 1 Hz were significant^17^. All the monitored patients our study had generalized slowing, and thirteen had generalized rhythmic delta activity.

Focal slowing on EEG has also been noted in patients with ICANS with focal neurologic symptoms such as aphasia. Studies with brain positron emission tomography (PET) confirm there are hypometabolic regions in these patients and the focal lesions can correlate with clinical symptoms^16^. Focal slowing is defined for lateralized theta and delta frequencies recorded over a hemisphere or a region which can be a sign of focal structural lesion or functional abnormality^22^. 5 patients in the B-ALL group had focal slowing over the following regions: right posterior quadrant, right frontal, left frontal, left frontotemporal, bilateral independent temporal slowing. 4 patients in the LBCL group had focal slowing over the following regions: 2 with left hemisphere slowing and 2 with left temporal slowing.

Seizures are a defining characteristic of high grade neurotoxicty and is part of ICANS evaluation criteria. Any type of seizure would place a patient into the high grade ICANS category (see supplemental Table 1). We found that seizures in ICANS could be of focal onset as well as generalized. Seizures were associated with increased morbidity, and patients were often intubated and sedated as part of treatment escalation^10^. Further complicating the diagnosis, clinical seizures on video EEG did not always have electrographic correlates, which may suggest patients can have surface negative seizures. We also described patients who developed non-convulsive seizures, confirming the need for continuous vEEG monitoring. Lastly, interictal epileptiform discharges (IED) were much less common compared to the number of seizures observed.

We observed a lower proportion of high-grade neurotoxicity as well as fewer seizures and epileptic discharges in patients with LBCL treated with commercial CAR products, compared with patients with B-ALL treated on our Phase I clinical trial. Factors that likely contributed to the high rates of overall seizures in the B-ALL patient cohort include differences in patient associated risk factors including disease type (B-ALL is not thought to be associated with higher risk for ICANS than LBCL), higher disease burden, and higher rates of severe CRS, among others. The patients with B-ALL treated with 1928z CAR T cells were also treated at a time when optimized toxicity management algorithms were not yet developed. Several patients also received cefepime or carbapenem antibiotics for febrile neutropenia during cytokine release syndrome which may have contributed to the increased rate of clinical seizures.

Seizure risk is greatest during the peak of ICANS symptomatology, and continuous monitoring can increase the yield for recording EEG abnormalities. Because of high risk of seizures in patients with severe ICANS, they likely require continuous video EEG monitoring for days until symptoms improve. To prevent seizures, the majority of institutions offer some form of prophylaxis, of which some are started at the onset of CAR T cell infusion while others are provided at the onset of CRS or ICANS^10^. In our study, despite levetiracetam treatment as seizure prophylactic approach, some patients eventually developed symptoms. As with other prophylactic approaches, anti-seizure medication should be carefully assessed by the treating physician, given the potential psychiatric adverse events associated to some anti-seizure drugs such as levetiracetam. However, given the small number of patients, larger focused studies in randomized patient cohorts should be conducted to weigh the efficacy and risks associated to anti-seizure prophylaxis. Similarly, future studies should prospectively address whether the use of prophylactic anakinra has any impact on seizure prevention.

EEG as a tool has some limitations: for instance, electrical activity on the surface of EEG encompasses inhibitory and excitatory postsynaptic potentials at the apical dendrites of cortical neurons. For a seizure or an IED to be detected by surface EEG electrodes, the activity needs to: (1) affect a large enough area of the brain; and (2) be close enough to the surface of the brain^23^. Given the frequent lack of interictal findings and potential of surface negative seizures, a longer EEG with video is preferred although evaluation should include at least a routine EEG.

Further, our study has some limitations, such as the retrospective scoring for the B-ALL cohort. We are also aware that ICANS severity can fluctuate over the course of a single day and even the prospective grading may not always match the exact EEG time with time of exam. Lastly, it was not feasible to conduct EEG in all patients who received CAR T cell therapy to compare EEG findings in patients that developed ICANS with those who did not. Another limitation of the study is that, given the lack of baseline assessment, future studies testing the utility of these imaging biomarkers should consider including them to robustly evaluate specificity. However, given our experience and the recent evidence from other institutions in comparable settings, our findings are likely generalizable to medical centers with vEEG capability and similarly trained neurophysiology staff. Future studies should focus on prospective validation and testing to determine the specificity and sensitivity of this approach to monitor ICANS in clinical and experimental settings.

## Electronic supplementary material

Below is the link to the electronic supplementary material.

Supplementary Material 1

## Data Availability

Data on patient seizures is provided within the supplementary information files. Detailed data include personal information that cannot be shared openly to protect study participant privacy. Clinical Data will only be shared using pooled analyses and individual-level data will not be publicly shared due to patient protection regulations. All the rest of deidentified materials and data will be shared following reasonable requests from qualified researchers. Requests should be addressed to DM.
