# Supplementary material for: EEG features and synek scale indicate severity of neurotoxicity in adult patients treated with CD19 CAR T-cell therapy
Source: Sci Rep. 2024 Nov 23;14:29090. doi: 10.1038/s41598-024-80566-0 (PMC11585625; doi:10.1038/s41598-024-80566-0)
Supplement: Supplementary file 1 — Supplementary Material 1 [file 41598_2024_80566_MOESM1_ESM.docx]

**Supplemental Table 1a.** ICANS grading: Adapted from ASTCT ICANS Consensus Grading for Adults. ICANS grade is determined by the most severe event. [12]

| **Neurotoxicity Domain** | **Grade 1** | **Grade 2** | **Grade 3** | **Grade 4** |
| --- | --- | --- | --- | --- |
| **ICE score** | 7-9 | 3-6 | 0-2 | 0 (patient is unarousable and unable to perform ICE) |
| **Depressed level of consciousness**^[†](https://www.sciencedirect.com/science/article/pii/S1083879118316914?via%3Dihub#tb6fn2)^ | Awakens spontaneously | Awakens to voice | Awakens only to tactile stimulus | Patient is unarousable or requires vigorous or repetitive tactile stimuli to arouse. Stupor or coma |
| **Seizure** | N/A | N/A | Any clinical seizure or nonconvulsive seizures on EEG that resolve with intervention | Life-threatening prolonged seizure (>5 min); or Repetitive clinical or electrical seizures without return to baseline in between |
| **Motor findings**^[‡](https://www.sciencedirect.com/science/article/pii/S1083879118316914?via%3Dihub#tb6fn3)^ | N/A | N/A | N/A | Deep focal motor weakness such as hemiparesis or paraparesis |
| **Elevated ICP/cerebral edema** | N/A | N/A | Focal/local edema on neuroimaging | Diffuse cerebral edema on neuroimaging; decerebrate or decorticate posturing; or cranial nerve VI palsy; or papilledema; or Cushing's triad |

**Supplemental Table 1b.** ICE Score [12]

| **ICE** |
| --- |
| • **Orientation:** orientation to year, month, city, hospital: 4 points • **Naming:** ability to name 3 objects: 3 points • **Following commands:** ability to follow simple commands: 1 point • **Writing:** ability to write a standard sentence: 1 point • **Attention:** ability to count backwards from 100 by 10: 1 point |

**Supplemental Table 2.** Patients with B-ALL treated with 1928z CAR who developed seizures.

|  | **Start of**  **ICANS** | **Clinical event description** | **Treatment** | **Electrographic correlate** | **Anti-seizure prophylaxis?** | **Non-convulsive Status epilepticus** |
| --- | --- | --- | --- | --- | --- | --- |
| B-ALL-1 | Post infusion day 11 | Day 11 - Witnessed GTC* seizure. | Lorazepam and fosphenytoin load given. | Did not capture further seizures. EEG showed diffuse slowing. | No | No |
| B-ALL-2 | Post infusion day 5 | Day 5 – Episode of aphasia Day 6 - myoclonic jerks  Day 7-Witnessed GTC seizure. | Fosphenytoin was added initially on day 5. On day 7, patient was given ativan and additional Fosphenytoin. Patient was intubated. | Did not capture further seizures. EEG showed diffuse slowing, triphasic waves and FIRDA. | No | No |
| B-ALL-3 | Post infusion day 7 | Day 7 – Bilateral jerks of arms and legs, Day 9 – Witnessed GTC seizure. | Initially no treatment for myoclonic jerks, then Fosphenytoin was given during GTC. | **EEG showed focal sharp waves and focal onset seizures**. | No | No |
| B-ALL-4 | Post infusion Day 9 | Day 11 - Witnessed GTC seizure. | Levetiracetam increased to 1000mg BID after seizure, intubated sedated after GTC | Did not capture further seizures. EEG showed diffuse slowing. | Levetiracetam 500 mg oral bid | No |
| B-ALL-5 | Post infusion  Day 12 | Day 7 - Lip smacking and staring, Day 14 - Witnessed GTC seizure | After GTC, lorazepam was given and Levetiracetam was increased. | **EEG showed generalized sharp waves with triphasic and biphasic morphology. These later progressed to generalized seizures.** | Levetiracetam 500 mg oral bid | Yes |
| B-ALL-6 | post infusion day 8 | Day 9 - Witnessed left arm shaking progressing to generalized tonic-clonic seizure during when sedation was weaned while patient was intubated. | Levetiracetam was increased to 1000mg BID after event. | **EEG showed focal sharp and generalized sharp waves, which were periodic. EEG also showed status epilepticus.** | Levetiracetam 500 mg oral bid | Yes |
| B-ALL-7 | Post infusion  day 8 | Day 8 - Witnessed generalized seizure activity for 30 seconds. | Levetiracetam was increased to 1000mg BID, no further seizures occurred. Patient was intubated. | Did not capture further seizures. EEG showed diffuse slowing. | Levetiracetam 500 mg oral bid | No |
| B-ALL-8 | Post infusion  day 10 | Day 10 - Patient had an ictal cry followed by witnessed GTC lasting over one minute. | Patient received lorazepam and was loaded with Fosphenytoin. Patient was intubated. | Did not capture further seizures. EEG showed diffuse slowing. | Already on Levetiracetam 1000 mg bid (prior provoked seizure) | No |
| B-ALL-9 | Post infusion  day 7 | Day 7 - Aphasia and had bilateral shaking. | Levetiracetam dose was increased to 1000mg BID. | Did not capture further seizures. EEG showed diffuse slowing and FIRDA. | Levetiracetam 500mg BID | No |
| B-ALL-10 | Post infusion  day 8 | Day 8 - Right arm and shoulder twitching. Then on day 9, had GTC seizure. | Levetiracetam dose was increased to 1000mg BID. | Did not capture further seizures. EEG showed diffuse slowing. | Levetiracetam 500mg oral BID | No |
| B-ALL-11 | Post infusion  day 5 | Day 5 – Patient developed expressive aphasia and then left foot and arm rhythmic jerking movements. | Lorazepam was given and levetiracetam was increased 1000mg BID. Intubated for airway compromise. | **Video captured an episode of arm shaking. EEG showed focal slowing and triphasic waves.** | Levetiracetam 500mg oral BID | No |
| B-ALL-12 | Post infusion Day 6 | Day 6 - Patient became aphasic and confused. Day 12 – Witnessed GTC | After GTC, Ativan given and Levetiracetam increased to 1000mg BID. Patient was intubated. | Did not capture further seizures. EEG showed diffuse slowing. | Levetiracetam 500mg oral BID | No |
| B-ALL-13 | Post infusion Day 9 | Day 9 - Witnessed GTC with post-ictal state. | Ativan and increase Levetiracetam a 1000mg BID. | Did not capture further seizures. EEG show diffuse slowing. | Levetiracetam 500mg oral BID | No |
| B-ALL-14 | Post infusion Day 9 | Day 9 - Episodes of confusion.  Day 10 - Had GTC seizure while photic stimulation was done for vEEG | Day 9 - Initially Levetiracetam was increased. Day 10 - After GTC, lorazepam and Fosphenytoin were given. Patient was intubated. Lacosamide added. | **Video captured right side clonic movements followed by tonic clonic activity. EEG showed focal onset seizure.** | Levetiracetam 500mg oral BID | No |
| B-ALL-15 | Day 7 | Day 9 - Patient became aphasic.  Day 11 - After disconnect of vEEG, patient had witnessed GTC. | Lorazepam was given, intubated for airway protection. Patient was loaded with Fosphenytoin. | **Video captured myoclonic jerks. EEG showed diffuse slowing, triphasic waves, and generalized rhythmic delta activity**. | Levetiracetam 500mg oral BID | Yes |
| B-ALL-16 | Day 8 | Day 10 - Patient had witnessed bilateral upper extremity shaking and a "frightened look". | Lorazepam was given. Levetiracetam was increased to 1000 then 1500 BID. | EEG initially showed diffuse slowing prior to seizure, then EMG artifact during seizure. Subsequently, there was burst suppression due to sedation | Levetiracetam 500mg oral BID | No |
| B-ALL-17 | Day 9 | Day 9 – Multiple witnessed GTC. | Given 4mg Ativan and Levetiracetam load. Levetiracetam was and increased to 1000mg BID. Patient was intubated. | **Video captured event with frequent blinking, then tonic-clonic movements of the left arm and subsequent spread to bilateral arms and legs. The EEG was obscured by myogenic artifact. There was diffuse delta and admixed theta.** | Levetiracetam 500mg oral BID | No |
| B-ALL-18 | Day 6 | Day 6 - Lip and hand twitching concerning for seizure | Continued levetiracetam 500mg BID | Did not capture further seizures. EEG shows diffuse slowing and FIRDA. | Levetiracetam 500mg oral BID | No |

*GTC – Generalized tonic-clonic seizure

**Supplemental Table 3.** LBCL patients with suspected seizures

|  | | **Start of ICANS** | **Description of event** | **Treatment** | **EEG findings** | **AED prophylaxis** | **Non-convulsive status epilepticus** |
| --- | --- | --- | --- | --- | --- | --- | --- |
| LBCL-1 | Post cell infusion day 3 | | Post infusion day 3 – involuntary movements and right facial twitching with bilateral eyelid fluttering | Given lorazepam and then loaded with Fosphenytoin. | Did not capture further seizures. EEG showed diffuse slowing and triphasic waves | Levetiracetam 500 mg oral bid | No |
| LBCL-2 | Post cell infusion day 5 | | Post cell infusion day 10 - sudden onset left arm flaccidity and dysarthria | Levetiracetam increased to 1000mg BID. | Did not capture further seizures. EEG showed diffuse slowing | Levetiracetam 500 mg oral bid | No |
| LBCL-3 | Post cell infusion day 6 | | Post infusion day 7 – Patient was agitated and not following commands | Loaded with Fosphenytoin and Levetiracetam increased to 1000mg BID. | EEG on day 7 showed centroparietal sharp waves and generalized sharp waves, which progressed to 1-2.5 hz generalized periodic discharges | Levetiracetam 500 mg oral bid | Yes |
| LBCL - 4 | Post cell infusion day 5 | | Post cell infusion day 5 – Right arm tonic posture and left arm with stereotyped movements (picking at her gown) | Loaded with Fosphenytoin and Levetiracetam increased to 1000mg BID | Did not capture further seizures. EEG showed bilateral slowing left>right. | Levetiracetam 500 mg oral bid | No |
| LBCL - 5 | Post infusion day 6 | | Post infusion day 6 and day 15 – Tremulous and not following commands | Keppra was increased to 1000mg BID | Did not capture further seizures. EEG showed left temporal slowing | Levetiracetam 500 mg oral bid | No |
